# Particle Swarm Optimisation: A Historical Review Up to the Current Developments

**DOI:** 10.3390/e22030362

**Published:** 2020-03-21

**Authors:** Diogo Freitas, Luiz Guerreiro Lopes, Fernando Morgado-Dias

**Affiliations:** 1 Madeira Interactive Technologies Institute (ITI/LARSyS/M-ITI), 9020-105 Funchal, Portugal; morgado@uma.pt; 2 Faculty of Exact Sciences and Engineering, University of Madeira, Penteada Campus, 9020-105 Funchal, Portugal; lopes@uma.pt

**Keywords:** Particle Swarm Optimisation (PSO), swarm intelligence, computational intelligence, bio-inspired algorithms, stochastic algorithms, optimisation

## Abstract

The Particle Swarm Optimisation (PSO) algorithm was inspired by the social and biological behaviour of bird flocks searching for food sources. In this nature-based algorithm, individuals are referred to as particles and fly through the search space seeking for the global best position that minimises (or maximises) a given problem. Today, PSO is one of the most well-known and widely used swarm intelligence algorithms and metaheuristic techniques, because of its simplicity and ability to be used in a wide range of applications. However, in-depth studies of the algorithm have led to the detection and identification of a number of problems with it, especially convergence problems and performance issues. Consequently, a myriad of variants, enhancements and extensions to the original version of the algorithm, developed and introduced in the mid-1990s, have been proposed, especially in the last two decades. In this article, a systematic literature review about those variants and improvements is made, which also covers the hybridisation and parallelisation of the algorithm and its extensions to other classes of optimisation problems, taking into consideration the most important ones. These approaches and improvements are appropriately summarised, organised and presented, in order to allow and facilitate the identification of the most appropriate PSO variant for a particular application.

## 1. Introduction

The Particle Swarm Optimisation (PSO) technique was proposed and initially developed by the electrical engineer Russell C. Eberhart and the social psychologist James Kennedy. The method was described in two papers [1,2] co-authored by those two authors and published in 1995, one of them having as its title the exact name of the technique they proposed.

This technique had (and still has) a deep connection with some social relations, concepts and behaviours that emerged from a computational study and simulation of a simplified social model of a bird flock seeking for food conducted by those authors, and it belongs to the so-called swarm intelligence, an important and extensive research area within natural computing.

The PSO method is based on the premise that the knowledge lies not only in the social sharing of information among generations but also between elements of the same generation. Although PSO has some characteristics that, in some sense and to a certain extent, have some similarity to those found in other population-based computational models, such as Genetic Algorithms (GA) and other evolutionary computing techniques, it has the benefit of being relatively simple, and its algorithm is comparatively easy to describe and implement.

In fact, its simplicity and apparent competence in finding optimal solutions in complex search spaces led the PSO algorithm to become well known among the scientific community, which contributed to its study and improvement. Thus, many approaches were suggested and different applications were tested with it, especially over the past decade. This review is intended to summarise all the main developments related to the PSO algorithm, from its original formulation up to current developments.

This review is organised as follows: Section 2 introduces the original PSO approach suggested by Eberhart and Kennedy [1,2]. Section 3 presents the most important parameter modifications and the main topological neighbourhood structures used with PSO. In Section 4, several PSO variants and its applications are presented. Subsequently, Section 5 introduces a number of hybrid algorithms resulting from combinations of PSO with other artificial intelligence tools. Finally, the last section presents some concluding remarks.

## 2. Particle Swarm Optimisation

The PSO computational method aims to optimise a problem iteratively, starting with a set, or population, of candidate solutions, called in this context a swarm of particles, in which each particle knows the global best position within the swarm (and its corresponding value in the context of the problem), along with its individual best position (and its fitness value) found so far during the search process in the problem’s solution space.

At each iteration, the velocity and the position of each particle in the swarm, represented by *d*-dimensional vectors, are influenced by the individual and the collective knowledge, which direct the repeated flights of the particles over the space of possible solutions to the problem in search of the optimum, until a suitable stopping criterion is satisfied.

The velocity of each particle *i* in the swarm, at every iteration *t*, is updated according to the following equation [3]:

(1) $${\vec{V}}_{\;t+1}^{\;i}={\vec{V}}_{\;t}^{\;i}+\varphi_{1}{R_{1}}_{t}^{i}\left({\vec{p}}_{\;t}^{\;i}-{\vec{x}}_{\;t}^{\;i}\right)+\varphi_{2}{R_{2}}_{t}^{i}\left({\vec{g}}_{\;t}-{\vec{x}}_{\;t}^{\;i}\right),$$

where $\varphi_{1}$ and $\varphi_{2}$ are real acceleration coefficients known respectively as cognitive and social weights, which control how much the global and individual best positions should influence the particle’s velocity and trajectory.

In the original PSO algorithm [2], both $\varphi_{1}$ and $\varphi_{2}$ are equal to 2, making the weights for the social and cognition parts, on average, equal to 1.

In multimodal problems, where multiple areas of the search space are promising regions, the fine-tuning of these parameters is even more critical to avoid premature convergence.

$R_{1}$ and $R_{2}$ are uniformly distributed *d*-dimensional random vectors, which are used to maintain an adequate level of diversity in the swarm population. Finally, ${\vec{p}}_{\;t}^{\;i}$ and ${\vec{g}}_{\;t}$ are, respectively, the personal or individual best position of particle *i* at iteration *t*, and the current global best position of the swarm.

In turn, the position of each particle *i*, at every iteration *t*, varies according to the following equation [3]:

(2) $${\vec{x}}_{\;t+1}^{\;i}={\vec{x}}_{\;t}^{\;i}+{\vec{V}}_{\;t+1}^{\;i}.$$

Note that ${\vec{x}}_{\;0}^{\;i}$ and ${\vec{V}}_{\;0}^{\;i}$ can be generated using a uniformly distributed random vector, whereas the particle’s best personal position should be initialised by its initial position; i.e., ${\vec{p}}_{\;0}^{\;i}={\vec{x}}_{\;0}^{\;i}$.

The information about the best personal position (and its fitness value) then flows through the imaginary connections among the swarm particles, making them move around in the *d*-dimensional search space until they find the best position that fulfils all the problem’s constraints.

These stochastic changes towards the ${\vec{p}}^{\;i}$ and $\vec{g}$ positions are conceptually similar to the crossover (or recombination) operation, which is the main exploration operation used by GA. However, in PSO, this operation is not necessarily applied by using a random probability.

The PSO algorithm has some advantages when compared to other continuous optimisation techniques; for instance: (i) it does not make assumptions on the continuity and differentiability of the objective function to be optimised; (ii) it does not need to compute the gradient of the error function; and (iii) it does not need good initial starting points or deep a priori knowledge about the most promising areas of the search space.

Besides that, PSO is a problem-independent algorithm; i.e., it can be used in a wide range of applications, since the only information that is needed to know to run the algorithm is the fitness evaluation of each candidate solution (and possibly the set of constraints of the problem).

The PSO algorithm has become better known over time, leading to other studies that extended its original formulation. Many variants have been suggested, such as the adoption of different communication structures (such as the use of ring and star topologies, often referred to as lbest models) as alternatives to the original approach (gbest model), wherein all particles are connected with each other [4,5,6].

### The Gbest and Lbest Models

A gbest model swarm, with *s* particles, is formally defined as:

(3) $${\hat{y}}_{\;t}\in \left\{\begin{array}{c} {\vec{p}}_{\;t}^{\;1},{\vec{p}}_{\;t}^{\;2},\ldots ,{\vec{p}}_{\;t}^{\;s} \end{array}\right\}|\;f\left({\hat{y}}_{\;t}\right)=\min\left(\left\{\begin{array}{c} f\left({\vec{p}}_{\;t}^{\;1}\right),f\left({\vec{p}}_{\;t}^{\;2}\right),\ldots ,f\left({\vec{p}}_{\;t}^{\;s}\right) \end{array}\right\}\right),$$

where $\hat{y}$ denotes the position of the best particle in the entire swarm or in its neighbourhood in a *d*-dimensional search space, also known as the target particle.

In this model, the information about the new positions found by any particle in the swarm is shared among all the others particles, which turns $\hat{y}$ into a kind of magnet, making all the particles converge to its position.

On the other hand, in a lbest model, the neighbourhood of size *l* of the particle *i* is defined as:

(4) $$N_{i}=\left\{\begin{array}{c} {\vec{p}}_{\;t}^{\;i-l},{\vec{p}}_{\;t}^{\;i-l+1},\ldots ,{\vec{p}}_{\;t}^{\;i-1},{\vec{p}}_{\;t}^{\;i},{\vec{p}}_{\;t}^{\;i+1},\ldots ,{\vec{p}}_{\;t}^{\;i+l-1},{\vec{p}}_{\;t}^{\;i+l} \end{array}\right\}.$$

Although this description of the lbest model assumes essentially a linear ordering of particles in a neighbourhood, which is not sufficiently generic, it is important to note that the neighbourhood may also use a two (or higher) dimensional topology.

The lbest model is formulated as below:

(5) $${\hat{y}}_{\;t}\in N_{i}\;|\;f\left({\hat{y}}_{\;t}\right)=\min\left(\left\{\begin{array}{c} f(\vec{a}) \end{array}\right\}\right),\;\forall \vec{a}\in N_{i}.$$

This means that, instead of sharing the information among all the particles in the swarm, the lbest model restricts the knowledge to the particles that are neighbouring each other. When *l* is set to be equal to *s*, the lbest model is equivalent to the gbest model.

The selection of the neighbourhood of each particle can be defined by each index *i*; however, it can be also defined by the distance between them. In this case, the set $N_{i}$ can be time-varying.

## 3. Modifications to the Particle Swarm Optimisation

Other different aspects of the original version of PSO have also been modified, and many variants have been proposed to address different kinds of problems; e.g., a discrete binary version of PSO [7] that is useful for combinatorial optimisation problems, such as the travelling salesman problem [8] and task scheduling problems [9,10].

Over time, PSO gained even more attention, and thus, more research was being done on it (see, e.g., [11,12] for an analysis of the trajectory and velocity of each particle during the execution of the PSO algorithm). This led many researchers to begin noticing problems with the original version of PSO, such as premature convergence (especially in multimodal domains) or performance issues (see, e.g., [13], wherein the number of fitness evaluations is reduced by using an estimated fitness value for each particle).

Premature convergence happens when some particle finds a local best position in the swarm that is not the global best solution to the problem. Other particles will then mistakenly fly towards it, without exploring other regions of the search space. In consequence, the algorithm will be trapped into that local optimum and will converge prematurely.

Many different approaches were suggested, and some were proven to be equivalent to the original PSO algorithm, leading to the same results. These changes were mainly in the population architecture and in the way of computing the next velocity of each particle in order to improve the efficacy and effectiveness of the search process and reduce the loss of diversity. In-depth studies were done to tune the parameters and to control velocity explosion (since the motion update equations usually tend towards infinity), stability and convergence [14].

### 3.1. Algorithm Convergence Improvements

#### 3.1.1. The Inertia Weight Parameter

In 1998, Shi and Eberhart [15] introduced the notion of the inertia weight, ω, of a particle. This coefficient controls the local and global search ability, determining how much influence the previous velocity should have on the current particle’s movement.

With this parameter, the velocity update equation (Equation (1)) is changed to:

(6) $${\vec{V}}_{\;t+1}^{\;i}=\omega {\vec{V}}_{\;t}^{\;i}+\varphi_{1}{R_{1}}_{\;t}^{\;i}\left({\vec{p}}_{\;t}^{\;i}-{\vec{x}}_{\;t}^{\;i}\right)+\varphi_{2}{R_{2}}_{\;t}^{\;i}\left({\vec{g}}_{\;t}-{\vec{x}}_{\;t}^{\;i}\right).$$

Most of the PSO algorithm variants developed since then include this coefficient. This is why the algorithm with this improvement is commonly referred to as the Standard PSO (SPSO).

Note that the original PSO velocity update equation can be obtained when ω=1.

Van den Bergh [16] stated a strong relationship between $\varphi_{1}$, $\varphi_{2}$ and ω, which can be modelled by the following inequality:

(7) $$\omega >\frac{1}{2}\left(\varphi_{1}+\varphi_{2}\right)-1.$$

When a high value is set for ω, the algorithm gives more importance to the particles’ self-knowledge, rather than the swarm’s knowledge (i.e., the other particles’ knowledge). On the other hand, a small inertia weight prevents the algorithm from converging to a local optimum, acting like a jumping out function. However, too many jumps will progressively worsen the algorithm’s properties, making it similar to a stochastic search [17].

As stated in [15], ω can be a positive constant (within the range [0.9,1.2]), but also a function of time (where time corresponds to the iteration number, *t*), or even a random number [18].

Unfortunately, and due to the lack of knowledge of the search process, it is difficult or impossible to develop a mathematical model to adjust the inertia weight dynamically [19]. Therefore, typically, to better control exploration and exploitation of the search space, ω is changed from 0.9 ($\omega_{\max}$) to 0.4 ($\omega_{\min}$) using a negative linear function of time [20,21], such as:

(8) $$\omega \left(t\right)=\omega_{\max}-\frac{\omega_{\max}-\omega_{\min}}{t_{\max}}\times t.$$

As Chatterjee and Siarry suggested [22], the inertia weight parameter can also be changed using a non-linear time-dependent function, such as:

(9) $$\omega \left(t\right)={\left(\frac{t_{\max}-t}{t_{\max}}\right)}^{n}\left(\omega_{\min}-\omega_{\max}\right)+\omega_{\max},$$

where $t_{\max}$ is the maximum number of iterations and *n* is the non-linear modulation index chosen by the user/researcher. According to those authors, n∈[0.9,1.3] is usually satisfactory.

Changing the particles’ momentum using a linear or a non-linear time-varying approach was proven to be the best rule of thumb in several applications, since the compromise between global and local searching throughout the course of the search process is critical to the success of the algorithm. That is, on its initials stages, the algorithm performs a fast initial exploration of the search space, but gradually becomes more focused around the best solution found until that point. This type of strategy is similar to the cooling schedule used in the Simulated Annealing (SA) algorithm.

Shi and Eberhart then suggested a fuzzy adaptive PSO algorithm [19] to better adapt the inertia weight to the search process. As the name suggests, a fuzzy system was implemented to improve the performance of the PSO by dynamically adjusting the inertia weight based on the global best position’s distance from an optimum.

In their benchmark tests, the fuzzy adaptive strategy was able to improve the performance of the PSO algorithm when compared with the use of a time-varying inertia weight parameter.

The PSO with inertia weight is considered a canonical PSO algorithm, since the search process runs iteratively in a region that is defined by each particle’s previous best position and velocity, the best previous successful positions of any of its neighbours and the particle’s current position.

#### 3.1.2. The Constriction Factor

In 1999, Maurice Clerc suggested the use of a constriction factor [6] to help the PSO algorithm solve optimisation problems faster, ensuring the convergence of the algorithm by making a trade-off between exploration and exploitation, affecting with this the particles’ trajectories around possible candidate solutions in the search space [6,23].

This constriction factor is given by:

(10) $$K=\frac{2}{\left|2-\varphi -\sqrt{\varphi^{2}-4\varphi }\right|},$$

where $\varphi =\varphi_{1}+\varphi_{1}$ and φ>4. Thus, Equation (1) may be written as:

(11) $${\vec{V}}_{\;t+1}^{\;i}=K\left[{\vec{V}}_{\;t}^{\;i}+\varphi_{1}{R_{1}}_{\;t}^{\;i}\left({\vec{p}}_{\;t}^{\;i}-{\vec{x}}_{\;t}^{\;i}\right)+\varphi_{2}{R_{2}}_{\;t}^{\;i}\left({\vec{g}}_{\;t}-{\vec{x}}_{\;t}^{\;i}\right)\right].$$

When the constriction factor is used with PSO, typically φ=4.1, and thus K≈0.7298.

Eberhart and Shi [20] compared the constriction factor with the inertia weight. These authors concluded that better quality solutions could be obtained with the constriction factor method, although mathematically the constriction factor and the inertia weight are equivalent, making the PSO with constriction factor and the SPSO equal when ω=K.

On the other hand, Eberhart and Shi [20] used the constriction factor while limiting the maximum velocity, since, when running a PSO algorithm without imposing restrictions to the velocities, these may rapidly increase within a few iterations to unacceptable levels, tending towards infinity. Basically, if ${\vec{V}}_{\;t+1}^{\;i}$ exceeds ${\vec{V}}_{\max}$ (defined by the user/researcher) in (11), then ${\vec{V}}_{\;t+1}^{\;i}={\vec{V}}_{\max}$.

${\vec{V}}_{\max}$ controls the global exploration ability of the swarm’s particles. Thus, if ${\vec{V}}_{\max}$ is too high, particles might overfly reasonable potential solutions (prioritising in this way the global exploration of the search space). However, if ${\vec{V}}_{\max}$ is too small, there will be diversity loss problems; that is, particles may not explore sufficiently the search space, and can be stuck in a local optimum.

Using five non-linear benchmark functions, those authors found that, when ${\vec{V}}_{\max}={\vec{x}}_{\max}$, the results improved significantly when compared with Clerc’s constriction factor *K*. However, there is a drawback: the need to know beforehand an approximation for the location of the global best position in order to limit $\vec{V}$.

Kar and his collaborators [24] combined the inertia weight parameter and the constriction factor to overcome the premature convergence and the stagnation problem (refer to Section 3.3), and thus improve the effectiveness and efficacy of the algorithm in a multidimensional search space. With this, the velocity is updated as follows:

(12) $${\vec{V}}_{\;t+1}^{\;i}=K\left[\omega {\vec{V}}_{\;t}^{\;i}+\varphi_{1}{R_{1}}_{\;t}^{\;i}\left({\vec{p}}_{\;t}^{\;i}-{\vec{x}}_{\;t}^{\;i}\right)+\varphi_{2}{R_{2}}_{\;t}^{\;i}\left({\vec{g}}_{\;t}-{\vec{x}}_{\;t}^{\;i}\right)\right].$$

It was reported by those authors that updating each particle’s velocity according to (12) produced better exploration and exploitation of the search space, along with faster convergence, for the test suite used.

**Figure 1:**
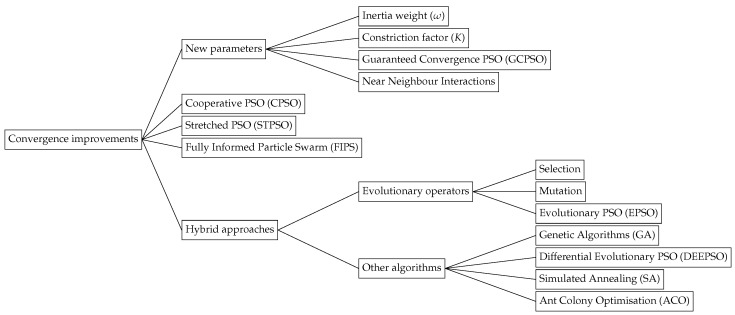
Summary of the most important convergence improvements developed for pso.

Convergence issues were the most reported problem related to the PSO algorithm. In order to lessen this problem, new parameters were introduced into PSO and different variants were suggested, including hybrid variants, as can be seen in Figure 1. Although some strategies to prevent premature convergence have not yet been mentioned, they were included in this figure for completeness. The reader is referred to the next sections for a description of the remaining approaches.

### 3.2. Neighbourhoods

#### 3.2.1. Static Neighbourhood

Simultaneously with the previously mentioned improvements in the PSO algorithm, some other different neighbourhood architectures were developed, in order to mimic the sociological phenomenon that an individual indirectly shares information with other people located around her/him.

In 1999, Kennedy reviewed and tested some of them [5], including circle/ring, wheel, star and random architectures. These are known as static architectures, because the neighbourhood does not change throughout the algorithm’s execution.

Because neighbourhood architectures produced different results when they were tested with different functions, the optimal pattern of connectivity between particles depended on the problem to be solved. For example, with a multimodal function, the wheel topology produced the best results, although star architectures performed better with unimodal functions.

Besides that, Kennedy [5] also concluded that the PSO with a large neighbourhood would perform better for simple problems, whereas small neighbourhoods should be used on complex problems.

Later on, in 2002, Kennedy and Mendes [25] synthesised all the population architectures developed so far: all-connected-to-all, pyramid, von Neumann, ring and star. They found that the best and the worst population architectures (based on consistency, performance, number of iterations and standard deviations from the known global best position) were, respectively, the von Neumann and the all-connected-to-all topologies (the last being the topology of the original PSO algorithm).

#### 3.2.2. Dynamic Neighbourhood

Meanwhile, Suganthan [26] proposed some improvements to the PSO algorithm, such as gradually increasing the local neighbourhood based on a computed radius for each particle.

If any particle is within the radius of another one, then they become neighbours and exchange information between them. As time goes by, this radius gradually becomes wider, until the swarm is fully connected.

The selection of the neighbourhood is, thus, based on the distance to each particle, rather than its indices, as occurs in the static neighbourhood topologies. These forms of neighbourhood organisation are called spatial topologies.

Suganthan [26] also suggested a gradual adjustment of the magnitude of the search in the search space by changing the values of the acceleration coefficients and the inertia weight during the course of the algorithm. Therefore, the parameters’ values are changed using the following equations:

(13) $$\begin{array}{cc} \omega & =\omega^{\infty }+\left(\omega^{0}-\omega^{\infty }\right)\;\left(1-t/t_{\max}\right),\\ \varphi_{1} & ={\varphi_{1}}^{\infty }+\left({\varphi_{1}}^{0}-{\varphi_{1}}^{\infty }\right)\;\left(1-t/t_{\max}\right),\\ \varphi_{2} & ={\varphi_{2}}^{\infty }+\left({\varphi_{2}}^{0}-{\varphi_{2}}^{\infty }\right)\;\left(1-t/t_{\max}\right), \end{array}$$

where the superscripts *∞* and 0 denote the final and the initial values of the parameters, respectively. In the tests carried out by this author, the initial value for ω was 0.95 and the final 0.2, whereas $\varphi_{1}$ and $\varphi_{2}$ had their values changed from 3 to 0.25 [26].

Suganthan [26] compared, for a set of test functions, his approach with the time-varying inertia SPSO algorithm ($\varphi_{1}$ and $\varphi_{2}$ were kept constant) and reported an improved performance when the parameters were changed according to (13).

In 2000, Kennedy proposed another approach for the lbest PSO, based on the spatial neighbourhood and on the ring neighbourhood topology, called social stereotyping [27].

The designation of this approach emerged, again, from social-psychological concepts, in this case the concept of stereotyping, where people are grouped according to, among other things, their social and physical characteristics, qualities, beliefs and opinions.

This social process often happens when people interact frequently with each other, becoming more and more similar, forming their opinions and making decisions based on the groups that they identify with.

As humans converge to the stereotypical behaviours and beliefs of the groups that they belong to, particles’ trajectories will be changed based on the region of the search space that they are in.

Each restricted search region of the search space is called a cluster. To constitute clusters in the search space, several particles are chosen as group leaders, called cluster centres or centroids. Then, the rest of the particles are grouped in a cluster based on the distance to each centre.

The PSO algorithm is modified so that the cognitive component (i.e., the previous individual particle’s best position) or the social component (i.e., the best previous position in the neighbourhood), or both, are replaced by the appropriate cluster centroid [16]. Thus, Kennedy [27] proposed three strategies to calculate the new velocity of each particle:

(14) $$\begin{array}{cc} {\vec{V}}_{\;t+1}^{\;i} & =\omega {\vec{V}}_{\;t}^{\;i}+\varphi_{1}{R_{1}}_{\;t}^{\;i}\left({\vec{c}}_{\;t}^{\;j}-{\vec{x}}_{\;t}^{\;i}\right)+\varphi_{2}{R_{2}}_{\;t}^{\;i}\left({\vec{g}}_{\;t}-{\vec{x}}_{\;t}^{\;i}\right),\\ {\vec{V}}_{\;t+1}^{\;i} & =\omega {\vec{V}}_{\;t}^{\;i}+\varphi_{1}{R_{1}}_{\;t}^{\;i}\left({\vec{p}}_{\;t}^{\;i}-{\vec{x}}_{\;t}^{\;i}\right)+\varphi_{2}{R_{2}}_{\;t}^{\;i}\left({\vec{c}}_{\;t}-{\vec{x}}_{\;t}^{\;i}\right),\\ {\vec{V}}_{\;t+1}^{\;i} & =\omega {\vec{V}}_{\;t}^{\;i}+\varphi_{1}{R_{1}}_{\;t}^{\;i}\left({\vec{c}}_{\;t}^{\;i}-{\vec{x}}_{\;t}^{\;i}\right)+\varphi_{2}{R_{2}}_{\;t}^{\;i}\left({\vec{c}}_{\;t}-{\vec{x}}_{\;t}^{\;i}\right), \end{array}$$

where ${\vec{c}}_{\;t}^{\;j}$ is the position of the centroid of the cluster *j* at the iteration *t*, and ${\vec{c}}_{\;t}$ is the centroid of the best particle selected from the neighbourhood.

Although it has a higher computational cost, and therefore, a longer execution time when compared to the original PSO, the first equation of (14) performed better than the standard velocity update equation.

#### 3.2.3. Near Neighbour Interactions

Veeramachaneni and his collaborators [28,29] proposed a simple, effective way to update each particle’s velocity dimension, motivated by the convergence behaviour issues detected in the PSO algorithm, especially in multimodal optimisation problems.

They developed an expression named Fitness-Distance-Ratio (FDR) that chooses the neighbourhood of each particle dimension based on the relative fitnesses of other particles in the neighbourhood:

(15) $$FDR=\frac{f\left({\vec{P}}_{\;t}^{\;j}\right)-f\left({\vec{x}}_{\;t}^{\;i}\right)}{|{\left({\vec{P}}_{\;t}^{\;j}\right)}_{d}-{\left({\vec{x}}_{\;t}^{\;i}\right)}_{d}|},$$

where ${\vec{P}}_{\;t}^{\;j}$ is the prior best position that maximises the FDR measure. Then, for each particle *i*, at every iteration *t*, each velocity dimension *d* is changed according to the following equation:

(16) $$\begin{array}{c} {\left({\vec{V}}_{\;t+1}^{\;i}\right)}_{d}=\omega {\left({\vec{V}}_{\;t}^{\;i}\right)}_{d}+\varphi_{1}{R_{1}}_{\;t}^{\;i}\left({\left({\vec{p}}_{\;t}^{\;i}\right)}_{d}-{\left({\vec{x}}_{\;t}^{\;i}\right)}_{d}\right)+\varphi_{2}{R_{2}}_{\;t}^{\;i}\left({\left({\vec{g}}_{\;t}^{\;i}\right)}_{d}-{\left({\vec{x}}_{\;t}^{\;i}\right)}_{d}\right)\\ \;+\varphi_{3}{R_{2}}_{\;t}^{\;i}\left({\left({\vec{P}}_{\;t}^{\;i}\right)}_{d}-{\left({\vec{x}}_{\;t}^{\;i}\right)}_{d}\right), \end{array}$$

where $\varphi_{3}$ is the deviation acceleration coefficient that corresponds to the importance, given by the particle, to the best experience of the best nearest neighbour.

Using this approach, besides the best position discovered so far, the velocity of each particle is also influenced by the previous positions visited by its neighbours.

Veeramachaneni et al. [28] reported that, although PSO performed well in the initial iterations of the benchmark test functions considered, overall results indicate that the FDR approach performed better in terms of convergence and thus in terms of the number of iterations.

**Figure 2:**
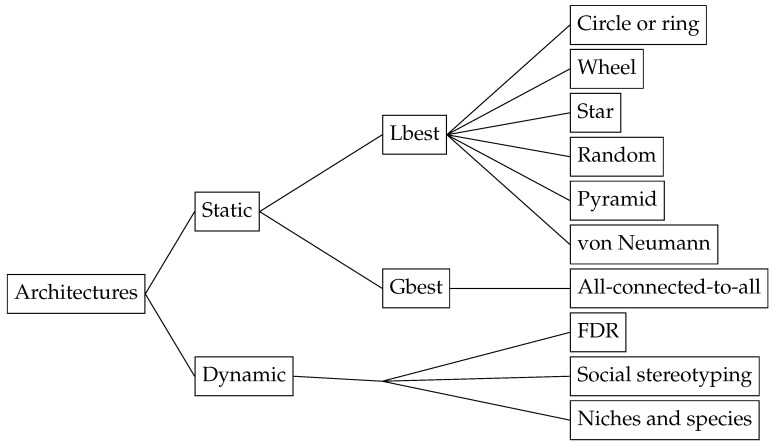
Summary of the most important architecture strategies developed for pso.

The different PSO architectures can be grouped into static neighbourhoods (in which the neighbourhood does not change during the execution of PSO) and dynamic neighbourhoods (where the neighbourhood changes according to, e.g., the number of iterations or the distance among particles in the search space), as shown in Figure 2. The reader is referred to Section 4.5.3 for a description of the niching and speciation strategies.

### 3.3. The Stagnation Problem

Van den Bergh [30] noticed a property that affected all gbest variants of the SPSO algorithm developed until then.

If a particle’s position is the same as the global best position, i.e., if ${\vec{x}}_{\;t}^{\;i}={\vec{p}}_{\;t}^{\;i}={\vec{g}}_{\;t}$, then the velocity in Equation (6) will only depend on $\omega {\vec{V}}_{\;t}^{\;i}$. This means that the particle will only leave this point if its previous velocity and ω are non-zero.

Otherwise, eventually all particles will stop moving, leading to premature convergence of the algorithm to a position that is not guaranteed to be the global best position or a local optimum, but only the best position so far found by the particles in the swarm. This problem is known as the stagnation problem.

To solve this problem, van den Bergh [30] proposed a new algorithm, called Guaranteed Convergence PSO (GCPSO), by inserting a new parameter τ into the SPSO algorithm, which denotes the index of the global best particle in the swarm.

Thus, the velocity and position update equations for the global best particle in the swarm are respectively changed by the following equations:

(17) $$\left\{\begin{array}{c} {\vec{V}}_{\;t+1}^{\;\tau }=-{\vec{x}}_{\;t}^{\;\tau }+{\vec{g}}_{\;t}+\omega {\vec{V}}_{\;t}^{\;\tau }+\rho \left(t\right)\left(1-2{R_{2}}_{\;t}^{\;i}\right),\\ {\vec{x}}_{\;t+1}^{\;\tau }\;={\vec{g}}_{\;t}+\omega {\vec{V}}_{\;t}^{\tau }+\rho \left(t\right)\left(1-2{R_{2}}_{\;t}^{\;i}\right). \end{array}\right.$$

The term $-{\vec{x}}_{\;t}^{\;\tau }$ resets the particle’s position to the global best position and $\omega {\vec{V}}_{\;t}^{\;\tau }$ sets the search direction; ρ(t) is a function that defines the diameter of the search area surrounding the global best position that will be randomly searched [31].

This significant change was used in several PSO variants (see, e.g., [31,32,33,34]).

However, on multimodal functions, the GCPSO algorithm has a higher probability of finding poor solutions when compared with PSO, due to faster convergence of the best particle towards a local extremum. Peer and his collaborators [35] studied this problem for the lbest models.

Nevertheless, as this situation is unlikely to occur, most of the authors do not consider this approach when updating the velocity and the position of the best particle in the swarm.

## 4. Particle Swarm Optimisation Variants

### 4.1. Cooperative Particle Swarm Optimisation

Due to the similarities between GA and PSO algorithms, some researchers started to propose PSO variants that combined the PSO algorithm with the operations used in GA.

An example of this is the Cooperative PSO (CPSO), a PSO variant proposed by van den Berg and Engelbrecht [36] and improved by the same authors [37]. The CPSO algorithm incorporates the concept of cooperation used in GA, wherein all subpopulations have to cooperate by contributing and exchanging information.

They suggested that this concept can also be applied to PSO by using a number of swarms for each dimension, instead of having only one for all dimensions. Thus, each subpopulation has only to optimise a 1-D vector. Although this approach seems simple, some changes on the original algorithm have to be made, especially to the evaluation of the objective function, which still requires a *d*-dimensional array as input.

Thus, a context vector was used to overcome the problem of the objective function evaluation. This vector is built at every iteration and has a component from each best particle’s dimension. Then, for each component, if the new value is better than the previous one, that specific component of the context vector is updated (and so the best individual fitness value).

The first variant splits the search space into exactly *d* subspaces [36]. On the other hand, and motivated by the fact that components may be correlated, in the $CPSO-S_{k}$ algorithm, proposed later by van den Bergh and Engelbrech [37], the search space is divided in *k* subspaces, where k≤d, which makes it a generalisation of the CPSO algorithm.

The $CPSO-S_{k}$ converges to the local optima of the respective subspaces, which makes it more propitious to be trapped in local optima. However, according to those authors, it has faster convergence when compared to PSO.

PSO, on the other hand, is more unlikely to be trapped in local optimum positions when compared with the $CPSO-S_{k}$ algorithm, because the optimisation process considers the dimensions as a whole.

Thus, $CPSO-H_{k}$, a hybrid approach using $CPSO-S_{k}$ and PSO, was suggested by van den Bergh and Engelbrech [37] to take the advantage of the proprieties of both algorithms, resulting in a fast one with an improved local escape mechanism.

In an overall assessment, the $CPSO-S_{k}$ and $CPSO-H_{k}$ algorithms perform better than PSO both in terms of quality of the solutions found and performance [37], especially when the dimensionality of the problem increases.

#### Two Steps Forward, One Step Back

Before getting into the details of the $CPSO-S_{k}$ and $CPSO-H_{k}$ [37] algorithms, van den Bergh and Engelbrecht [36] stated one problem with PSO, which they named two steps forward, one step back. They found that, at each iteration, PSO changes the elements of the *d*-dimensional vector, making some components move close to the optimal solution, although others can move away from it. Thus, PSO can accept a new candidate solution if its fitness value is lower than the previous one (when considering minimisation problems).

In their paper, they showed an example of this weakness of PSO with a vector with three components, wherein one component already had the optimal value, but its value changed in the next iteration to a poor one. Despite that, the other two components improved, and so did the fitness value.

In this case, two components improved, although one did not, taking the algorithm two steps forward and one step back. To overcome this problem, van den Bergh suggested evaluating the fitness function as soon as a component changes, while keeping constant the other d−1 components with the values of the previous iteration.

### 4.2. Adaptive Particle Swarm Optimisation

In 2009, one important approach for solving both unimodal and multimodal functions effectively, as well as improving the search efficacy and the converge speed of PSO while preserving premature convergence, was proposed by Zhan et al. [38].

The Adaptive PSO (APSO) presented by those authors defines four evolutionary states for the PSO algorithm: exploration, exploitation, convergence and jumping out, according to the evaluation of the swarm’s distribution and each particle’s fitness. Thus, for each state, different strategies can be applied, such as parameter adaptation.

The swarm’s distribution can be assessed by the mean distance of each particle to all other particles using the following Euclidean metric:

(18) $$D_{\;t}^{\;i}=\frac{1}{s-1}\sum_{j=1,j\neq i}^{s}\sqrt{\sum_{i=1}^{d}{\left({\vec{x}}_{\;t}^{\;i}-{\vec{x}}_{\;t}^{\;j}\right)}^{2}},$$

where *s* is the size of the swarm and *d* is the number of dimensions.

Then, an evolutionary factor, $e_{f}$, is computed by:

(19) $$e_{f}=\frac{D_{g}-D_{\min}}{D_{\max}-D_{\min}}\in \left[0,1\right],$$

where $D_{\max}$ and $D_{\min}$ are respectively the maximum and minimum distances among the particles, and $D_{g}$ is the value of $D_{\;t}^{\;i}$ of the globally best particle in the swarm.

Based on this factor, the algorithm can be then classified in one of the evolutionary states. For example, a medium to substantial value of $e_{f}$ indicates the exploration state, while a shrunk value of $e_{f}$ means exploitation. In turn, the convergence state happens when a minimum value of $e_{f}$ is reached, and the jumping out state when the mean distance value for the best particle is significantly higher than the mean distance value for the other particles.

An adaptive $e_{f}$-dependent inertia weight was also suggested by the same authors and is given by:

(20) $$\omega \left(e_{f}\right)=\frac{1}{1+1.5e^{-2.6e_{f}}}\in \left[0.4,0.9\right],\;\forall \;e_{f}\in \left[0,1\right].$$

Thus, when $e_{f}$ is large (jumping out or exploration state), $\omega (e_{f})$ makes the algorithm give more importance to the particle’s self-knowledge, thereby benefiting the global search. On the other hand, when $e_{f}$ is small (exploitation or convergence state), the swarm’s knowledge is more relevant than the self-knowledge of each particle, giving priority to the local search.

The cognitive and social weights are also changed, according to the evolutionary state, and a Gaussian mutation operation is applied to the best particle in the swarm to enable it to jump out of a local optimum or to refine the global best solution.

If the new position found is better than the best particle’s solution, the new one replaces the best particle’s position. Otherwise, the worst particle’s solution is replaced by this new position.

The velocity and the position of each particle are computed, and as usual, the PSO algorithm keeps iterating until the stopping criterion is met.

When tested with some unimodal and multimodal functions, APSO showed itself to be efficient at improving the convergence speed, and most importantly, at enhancing the accuracy of the algorithm when compared to other well-known approaches.

### 4.3. Constrained Optimisation Problems

On the other hand, Parsopoulos and Vrahatis [39] proposed a method based on a penalty function and on the constriction factor for constraint handling with PSO. To the authors’ best knowledge, this was the first paper that proposed a method to use PSO to optimise constrained optimisation problems.

A Constrained Optimisation Problem (COP) can be transformed into an unconstrained problem by using a penalty function that penalises the objective function if the conditions on the variables are not held. Therefore, a single objective function is built and optimised using a standard unconstrained optimisation algorithm.

A penalty function, $f(\vec{x})$, can be defined as:

(21) $$f\left(\vec{x}\right)=f\left(\vec{x}\right)+h_{p}\left(t\right)H_{p}\left(\vec{x}\right),\;\;\vec{x}\in S\subseteq R^{d},$$

where $f(\vec{x})$ is the original objective function to be optimised, $h_{p}\left(t\right)$ is a dynamic modified penalty value, and $H_{p}\left(\vec{x}\right)$ is the penalty factor defined as:

(22) $$H_{p}\left(\vec{x}\right)=\sum_{i=1}^{m}\;\theta \left(q_{i}\left(\vec{x}\right)\right)\;q_{i}{\left(\vec{x}\right)}^{\gamma (q_{i}\left(\vec{x}\right))},$$

where $q_{i}\left(\vec{x}\right)=\max\left(\left\{0,g_{i}\left(\vec{x}\right)\right\}\right)$ for i=1,…m, $\theta \left(q_{i}\left(\vec{x}\right)\right)$ is a multi-stage assignment function, and $\gamma \left(q_{i}\left(\vec{x}\right)\right)$ is the power of the penalty function. Note that although the equality constraints $h_{i}$ were not considered, they can be transformed into two inequality constraints, such as $g_{i}\left(\vec{x}\right)\leq 0$ and $-g_{i}\left(\vec{x}\right)\geq 0$.

Although COPs can be transformed into unconstrained problems by using a penalty function, they require more parameters to be fine-tuned (in this case, $h_{p}\left(k\right)$, $\theta \left(q_{i}\left(\vec{x}\right)\right)$ and $\gamma \left(q_{i}\left(\vec{x}\right)\right)$) in order to prevent premature convergence.

Hu and Eberhart [40,41] proposed a more straightforward, brute-force method to optimise COPs, known as the Preservation of Feasible Solutions Method (FSM).

In their proposal, all feasible solutions found during the search process in the whole search space are preserved. After a stopping criterion is met, the optimal solution that fulfils all the problem’s constraints may be found.

When these two methods are compared using the same problems, fine-tuning of the penalty function parameters may result in better average optimal solutions when compared to FSM, but the choice of which constraint handling method to be used may be very problem-dependent [42].

He et al. [43] introduced into PSO a different constraint handling method, called fly-back mechanism. The idea is simple: when a particle fly to a non-feasible region of the search space, its position is reset to the previous (feasible) position.

On the other hand, Sun et al. [44] proposed a more advanced approach, in which once a particle enters a non-feasible region, a new feasible position is computed by:

(23) $${{\vec{x}}^{\prime }}_{\;t+1}^{\;i}={\vec{x}}_{\;t}^{\;i}+\alpha {\vec{V}}_{\;t+1}^{\;i},$$

where the coefficient α is a diagonal matrix whose diagonal values are set within the range of [0,1]. Thus, if $\alpha_{ii}=1$ for i=1,…,d, then this means that ${\vec{x}}_{\;\;t+1}^{\;\;i}$ is a feasible position.

If ${\vec{x}}_{\;\;t+1}^{\;\;i}$ is not in a feasible position, α must be adjusted to bring the particle back to a feasible position.

Sun et al. [44] suggest that α should be found by:

(24) $$\min\left(\prod_{k=1}^{m+2d}e^{\max(0,\;g_{k}\left({\vec{x}}_{\;t}^{\;i}+\alpha {\vec{V}}_{\;t+1}^{\;i}\right))}\;\prod_{j=1}^{p}\;e^{\max(0,\;\mathrm{abs}\left(h_{j}\left({\vec{x}}_{\;t}^{\;i}+\alpha {\vec{V}}_{\;t+1}^{\;i}\right)\right)}\right).$$

Note that the superscript m+2d on the first product symbol includes both the number of inequality constraints, as well as the search space’s boundaries, that are transformed into two inequality constraints.

Then, the algorithm proceeds like the PSO algorithm until a stopping criterion is met.

Results show that this algorithm is suitable for solving COPs. However, it did not perform as well when the optimal values were at the boundaries of the search space.

### 4.4. Multi-Objective Optimisation

Initially, research on PSO was made considering only the optimisation of one function. However, in real-world problems, it is rare to have only a single objective to optimise, but multiple objectives that should be optimised simultaneously.

At first glance, the different functions can be optimised running the algorithm independently for each of them, but optimal solutions seldom are found, because the objectives may conflict with each other (e.g., price–quality relationship).

The multi-objective optimisation problems can be modelled as finding $\vec{x}\in S\subseteq R^{d}$ that minimises $f\left(\vec{x}\right)={\left[\begin{array}{c} f_{1}\left(\vec{x}\right),f_{2}\left(\vec{x}\right),\ldots ,f_{k}\left(\vec{x}\right) \end{array}\right]}^{T}$.

In most of the multi-objective optimisation problems, there is no single solution that simultaneously optimises each objective but a set of feasible solutions called Pareto optimal solutions, $\vec{y^{*}}$. In other words, there is no feasible vector $\vec{x}$ that would optimise some objective values without penalising at least one other objective value.

This set of feasible solutions forms the so-called Pareto front. The user/researcher is then responsible for choosing what he considers to be the best solution to the problem at hands.

This introduces a notion of dominance, called Pareto Dominance: a vector $\vec{u}=\left[u_{1},u_{2},\ldots ,u_{k}\right]$ is said to dominate $\vec{v}=\left[v_{1},v_{2},\ldots ,v_{k}\right]$ if $\forall i\in \left\{1,2,\ldots ,k\right\},u_{i}\leq v_{i}\wedge \exists i\in \left\{1,2,\ldots ,k\right\}:u_{i}<v_{i}$.

Hu and Eberhart [45] proposed an approach to solving multi-objective optimisation problems with a PSO algorithm based mainly on the concept of Pareto optimally.

They presented a dynamic neighbourhood version of PSO, such that, at every iteration, each particle has a different neighbourhood than it had in the previous iteration.

Each particle’s neighbourhood is chosen based on the distances from the current particle to the other particles in the fitness value space of the first objective function to be optimised.

Within its neighbourhood, each particle chooses the local best (lbest) particle, considering the fitness value of the second objective function.

The new ${\vec{p}}_{\;t}^{\;i}$ is only set when a new solution that dominates the current ${\vec{p}}_{\;t}^{\;i}$ is found.

Unfortunately, Hu and Eberhart only used two objective functions to describe their proposal and did not provide enough details on how the algorithm was implemented, especially regarding how to compute the distance between particles. Besides that, their proposal, in essence, only optimises one objective function, and nothing guarantees that the optimal solution for the second function is also the optimal solution for the first one.

Coello Coello and his collaborators [46,47], on the other hand, introduced the notion of external (or secondary) repository, proposing a PSO variant called Multi-Objective PSO (MOPSO). The external repository stores non-dominated vectors of particles’ positions used to compute the velocity of each particle at each iteration (replacing ${\vec{g}}_{\;t}$ in (6)). This repository is dynamically chosen within each iteration. For example, if none of the elements contained in the external population dominates the new solution found, then such solution is stored in the external repository. They also used a constraint handling mechanism to solve multi-objective constraint optimisation problems with PSO, and a mutation operator to ensure the diversity of the particles, to slow down the convergence speed and to prevent premature convergence to a local optimum.

The constraint handling mechanism can do one of two things if a particle goes beyond the boundaries: either set it to its corresponding boundary, or multiply its velocity by −1 in order to search in the opposite direction.

According to a certain probability, a mutation operator is applied to only one randomly chosen dimension of each particle by changing its value according to the current and total number of iterations, taking into account its boundaries, however. This was the first mutation operation proposed to solve optimisation problems with constraints using PSO.

The algorithm then proceeds as the standard PSO until a stopping criterion is met. The output of the algorithm is a Pareto front, which is built upon each iteration as a grid using the values of the external repository.

The MOPSO approach showed better results than other multi-objective evolutionary algorithms and required low computational time to run the algorithm.

These approaches were the first steps of the research on solving multi-objective parameter optimisation problems using PSO. The MOPSO algorithm was improved by Fieldsend [48] and later by Mostaghim [49].

### 4.5. Multimodal Function Optimisation

Simultaneously, efforts were made to extend the PSO algorithm for multimodal function optimisation; that is, for finding all the global best positions (and eventually other local optimal solutions) of an equation or system of equations.

This type of optimisation is especially useful for the decision makers, so that decisions can be made taking into account, for example, physical and cost constraints, having, however, multiple optimal solutions at hand.

Due to the existence of multiple local and global optima, all these problems can not be solved by classical non-linear programming techniques. On the other hand, when using Evolutionary Algorithms (EA) and PSO, the optimum positions can be found faster than by traditional optimisation techniques [50].

However, PSO was designed to find only one optimum of a function, and so some changes are required. In fact, PSO can be applied multiple times on the same function to find all the desired minima. Nevertheless, it is not guaranteed that all will be found.

In this type of optimisation, fast convergence can sometimes lead to premature convergence, because PSO (or other evolutionary algorithms) may get trapped into local optima. Thus, it is important to maintain the population diversity before some goal is met.

At first glance, the lbest models can be thought of as potential candidates to find multiple solutions, in which each neighbourhood will represent a candidate solution. However, one particle can be in several neighbourhoods at the same time, causing all the particles in these neighbourhoods to converge to the same point in case that particle has the best fitness among all the points in the neighbourhoods it belongs to. Consequently, if that point is a local optimum, these neighbourhoods will be biased towards that position, making the algorithm converge prematurely.

Thus, many approaches to tackling this kind of problem have been suggested, and the most relevant will be described in the next subsections.

#### 4.5.1. Objective Function Stretching

Multimodal function optimisation with PSO was first introduced by Parsopoulos et al. [50]. The first version of their algorithm, known as Stretched PSO (STPSO), had the main objective of finding a global minimum of a multimodal function, avoiding the algorithm being trapped in local optima.

To do so, they defined a two-stage transformation on the objective function that is applied to it as soon as a local optimum (minimum) is found, using a function stretching technique.

A function stretching ($H(\vec{x})$) acts like a filter, transforming the form of the original function in a more flatter surface yet highlighting possible global and local optimum positions.

As already said, this transformation is applied as soon as a local minimum is found, in order to repel the rest of the swarm from moving towards that position. After that, $f(\vec{x})$ is replaced by $H(\vec{x})$ and the PSO algorithm is applied until a specific stopping criterion is met.

Parsopoulos and Vrahatis [51] extended this approach to find all globally optimal solutions and showed that this new approach could be effective and efficient.

They defined a threshold, ϵ, related to the requested accuracy so that when the value of the objective function applied to the particle is lower than ϵ, this particle is pulled away from the swarm and a function stretching is applied at that point to avoid the rest of the swarm from moving towards that position.

After this transformation, a new particle is randomly added to the swarm, to replace the one that was isolated from it. Then, if the function value of the isolated particle is higher than the desired accuracy, a new sub-swarm is created (which is considered a niching technique), and a new instance of the algorithm is executed, although being conditioned to that search area.

The algorithm stops when the number of global minimisers reaches a known one, or when the number of global minimisers is unknown, at the maximum number of iterations.

Unfortunately, this stretching transformation (that can also be considered as a convergence acceleration technique) may create local minima that were not present in the original objective function. This may require some restarts of the PSO algorithm until a global minimum is found [52].

Thus, Parsopoulous and Vrahatis [53] improved their method again by introducing deflection (a technique that incorporates knowledge from previously detected minimisers into the objective function) and a better repulsion technique (which ensures that if a particle moves towards one of the detected local optima, it will be repelled away from it).

#### 4.5.2. Nbest Technique

In 2002, Brits et al. [52] proposed a new PSO-based technique, known as neighbourhood best or nbest PSO, and showed its successful application in solving systems of unconstrained equations.

A system of equations with *k* equations can be transformed into one fitness function:

(25) $$f\left(\vec{x}\right)=\sum_{i=1}^{k}\left|f_{i}\left(\vec{x}\right)\right|,$$

where each equation is algebraically rewritten to be equal to zero. However, the formulation of the problem using this transformation fails when multiple solutions are present in the search space.

To overcome this problem, they redefined the objective function as the minimum of the fitness function with respect to other equations. That is, as in the example given by Brits and his collaborators, when a system of equations has three equations (*A*, *B* and *C*), the objective function is defined as the minimum of the combinations of those equations:

(26) $$f\left(\vec{x}\right)=\min\left(\left\{f_{AB}\left(\vec{x}\right),f_{AC}\left(\vec{x}\right),f_{BC}\left(\vec{x}\right)\right\}\right).$$

Thus, particles that are close to one of the solutions are rewarded and do not suffer any penalisation if they are still far from the global best particle.

The nbest technique uses a dynamic neighbourhood approach, based on the Euclidean distance between the particles, to change the biased information towards a single optimal solution.

It is noteworthy that the Euclidean distance is computationally intensive to calculate, and besides that, choosing the neighbourhood based on it led to undesirable convergence properties. Thus, later, Euclidean neighbourhood was abandoned.

After computing the Euclidean distance from each particle to each other one, the neighbourhood of each particle is defined and the centre of mass of the positions is kept as neighbourhood best, and the PSO algorithm proceeds normally until a stopping criterion is met.

The results presented by those authors showed that the nbest technique can find all globally best solutions. However, in real-world applications, the systems of equations to optimise are usually not limited to three equations, and frequently the number of them is much higher. Thus, in such cases, this solution may face performance issues as the number of combinations can increase rapidly.

#### 4.5.3. Subpopulations and Multi-Swarm

Another strand for the neighbourhood structure of communication happens when some subpopulations are watching over the best local optimum. That is, when a local optimum is found, the original swarm is split. One fraction of the swarm remains to explore the local optimum, and the other continues the search on a different portion of the search space [54].

In natural ecosystems, animals live and reproduce in the same groups of their own species, called niches. Based on this idea, niching techniques were proposed and implemented successfully with GA and latter with PSO.

This type of technique is most commonly used in multimodal search spaces, because groups of individuals can move simultaneously into different search space regions. Note that individuals can be grouped by similar fitness values, by their distance from others or other similarity criteria.

Brits et al. [31] suggested the first PSO niching technique, named NichePSO, for successfully locating multiple optimal solutions in multimodal optimisation problems simultaneously.

In their proposal, they used a main swarm and a number of sub-swarms, as well as two variants of the PSO algorithm, namely, GCPSO [30] and the cognition-only model proposed by Kennedy [55], where Equation (1) is changed to only include the cognitive weight; i.e.,

(27) $${\vec{V}}_{\;t+1}^{\;i}={\vec{V}}_{\;t}^{\;i}+\varphi_{1}{R_{1}}_{\;t}^{\;i}\left({\vec{p}}_{\;t}^{\;i}-{\vec{x}}_{\;t}^{\;i}\right),$$

thereby allowing each particle to perform a local search, preventing the situation in which all particles get pulled towards a single solution due to the influence of the best particle or particles in the neighbourhood.

The cognition-only PSO variant is run for one iteration in the main swarm. Particles are then grouped by a given accuracy threshold (similar to the one used by Parsopoulos and Vrahatis [39] in the constriction factor PSO approach), and then, for each sub-swarm, GCPSO is run.

After that, the sub-swarms that are too close can be merged and can absorb particles from the main swarm when they move into them. Finally, the algorithm checks in the main swarm for the need to split it in other swarms, and iterates until a stopping criterion is found (for example, when it reaches a certain number of globally optimal solutions, when known).

Later, Engelbrecht [34] improved the NichePSO by changing the merging and absorption strategies that were proposed in the original approach. Schoeman and Engelbrecht [56] proposed a PSO approach (which can be considered as a sequential niching PSO) that uses an additional vector operation, namely, the dot product, to change the direction in which particles should be headed to; viz., towards an already located niche or to explore and search for a new niche. Shortly after that, the same authors [57] proposed a parallel vector-based approach wherein all particles are updated simultaneously.

Li [58] extended the FDR-PSO algorithm to multimodal optimisation problems by introducing two mechanisms in the original FDR-PSO: the memory-swarm and the explorer-swarm.

The memory-swarm saves the personal best positions found so far by the population. During its turn, the explorer-swarm saves the current state of the particles and is used to explore the search space.

The best positions in the memory-swarm are used as anchors, and as the algorithm runs, niches are created around the best positions, according to the fitness-Euclidean distance ratio between a particle’s personal best and other personal bests of the particles in the population.

The fitness-Euclidean distance ratio technique is an improved version of FDR that has a scaling factor computed using the worst and best fitted particles in the swarm.

Li et al. [59] split the population into species, according to the distances between the particles. Based on this idea and the ideas presented in [60,61], Parrott and Li [62] incorporated the concept of speciation into the constriction factor approach of PSO for solving multimodal optimisation problems.

It is important to note that, although different terminology is used, both niching and speciation techniques group similar particles by a given criteria.

In the resulting species-based algorithm, the particles are dynamically and adaptively grouped into species around dominating particles called species seeds, each species being used to track an optimum point.

Li [63] also presented a niching, parameter-free algorithm with ring topology for multimodal optimisation, which is able to form stable niches across different local neighbourhoods.

Four variants of this lbest PSO niching algorithm with ring topology were also suggested by Li [63], two of them (r2pso and r3pso) with an overlapping ring topology—the other two variants, namely, r2pso-lhc and r3pso-lhc, being lbest PSO algorithms with a non-overlapping ring topology.

Recently, Yue et al. [64] improved the lbest PSO niching algorithm by including a Special Crowding Distance (SCD) for solving multimodal multi-objective problems and reported that the algorithm was able to find a more significant number of Pareto-optimal solutions when compared to other well-known algorithms.

### 4.6. The Fully Informed Particle Swarm Optimisation

In 2004, Mendes et al. [65] introduced the Fully Informed Particle Swarm (FIPS) optimisation algorithm, because they were convinced that each particle should not be influenced only by the best particle among its neighbours, but all the neighbours must contribute to the velocity adjustment of each particle; i.e., the particles should be fully informed.

They integrated the constriction factor approach of PSO with a new velocity update equation, wherein the social component is not explicitly considered, given by:

(28) $${\vec{V}}_{\;t+1}^{\;i}=K\left({\vec{V}}_{\;t}^{\;i}+\varphi \;\left({\vec{p}}_{\;t}^{\;i}-{\vec{x}}_{\;t}^{\;i}\right)\right).$$

Typically φ=4.1 and K≈0.7298. The particle’s individual best position ${\vec{p}}_{\;t}^{\;i}$ is given by:

(29) $${\vec{p}}_{\;t}^{\;i}=\frac{\sum_{\;i=1}^{\;l}\sigma \left(i\right)\;{\vec{\varphi }}_{i}\times {\vec{p}}_{\;t}^{\;i}}{\sum_{\;i=1}^{\;l}\sigma \left(i\right)\;{\vec{\varphi }}_{i}},$$

with

(30) $${\vec{\varphi }}_{i}=\vec{U}\left[\begin{array}{c} 0,\frac{\varphi_{\max}}{l} \end{array}\right]\;,\;\forall i\in \left\{1,\ldots ,l\right\},$$

where *l* is the number of particles in the population, and $\vec{U}$ is a function that returns a position vector generated randomly from a uniform distribution between 0 and $\varphi_{\max}/l$.

The function σ(i) can return a constant value over the iterations, or as Mendes et al. [65] also did in their experiments, return the fitness value of the best position found by the particle *i* or the distance from that particle to the current particle.

Although in this variant all particles contribute equally for the change in the next velocity calculation, those authors also suggested a weighted version of the FIPS algorithm, in which contributions are given according to the fitness value of the previous best position or the distance in the search space to the target particle.

They were in fact right, since both FIPS variants performed well on the considered neighbourhood architectures (except on the all-connected-to-all), finding at all times the minimum of the benchmark functions. The weighted versions require an extra computational cost, and such cost may not be justified, since the unweighted version performed quite well in their study [65].

### 4.7. Parallel Implementations of Particle Swarm Optimisation

Besides being trapped into local optima, PSO has another problem: its performance becomes progressively worse as the dimensions of the problem increase [66]. To alleviate this problem, some approaches were suggested, such as the use of multiple processing units of a computer system to distribute processing among them, creating sub-swarms, and thus speeding up the execution of the algorithm.

As each sub-swarm can be thought to be independent, PSO maps well to the parallel computing paradigm. In this section, a survey of the most common approaches to Parallelized PSO (PPSO) will be described.

For PPSO approaches, a multi-core Central Processing Unit (CPU) or a Graphics Processing Unit (GPU) can be used to process the tasks of each parallel sub-swarm, along with some mechanism to exchange information among them. The exchange of information can be made in a synchronous or asynchronous manner.

Synchronous exchange is made when particles of each sub-swarm are synchronised with each other, i.e., the particles wait for the others to move to the next iteration, leading to the same result as the sequential approach, although its processing is done in parallel. On the other hand, when the exchange of information is made asynchronously, the sub-swarms are independent of each other, and thus, at the end of an iteration, each particle uses the information available at the moment (especially the global best position information) to move to the next position.

In addition, different architectures can be used to control the exchange of information, such as master–slave (where there is one processing unit that controls the execution of the other processing units), fine-grained (in which the swarm is split into sub-swarms and arranged in a 2-D grid, wherein the communication is only made within the neighbours of each sub-swarm) and coarse-grained (where the swarm is also split into sub-swarms independent of each other; however, from time to time, they exchange particles between them) [23,66,67].

Gies and Rahmat-Samii [68] proposed the first PPSO. They reported a performance gain of eight-fold (when compared with sequential PSO) with the PPSO algorithm for finding the optimal antenna array design. The results of this first work about PPSO motivated other researchers, such as Baskar and Suganthan [69], who improved the performance of FDR-PSO [29] by introducing a novel concurrent approach, called CONPSO.

Three communication strategies were presented in [70,71] by using the GA’s migration technique to spread the gbest position of each sub-swarm to the others. In the first one, the best particle of each sub-swarm is mutated and migrated to another sub-swarm to replace the poorest candidate solutions. In the second strategy, on the other hand, although similar to the previous one, the exchange of information only happens in neighbour sub-swarms. Finally, the latter solution is a hybrid between the first and the second strategy.

Schutte et al. [72,73] used a synchronous master-slave architecture for a bio-mechanical system identification problem. All particles were evaluated using parallel processes; however, all processes had to finish in order to update the next velocities and positions of all particles. Additionally, they reported that the time required to solve the system identification problem considered was reduced substantially when compared with traditional approaches.

As stated by Schutte et al. [73], synchronous implementations of PPSO are easy to produce. Nevertheless, such implementations usually have a poor parallel efficiency, since some processing units may be idle. Due to this fact, Venter and Sobieszczanski-Sobieski [74] proposed a master–slave asynchronous implementation PPSO algorithm and compared it with a synchronous PPSO.

One can consider the fact that the behaviour of each particle depends on the information available (possibly not from all other sub-swarms) at the start of a new iteration as a drawback of asynchronous approaches. However, in the authors’ opinion, this can be negligible because, although particles may not have updated information about the best solution before moving to a next position in the search space, communication always exists between particles and sub-swarms. Thus, in further iterations, the information about the best position found so far will inevitably be shared.

Koh et al. [75] introduced a point-to-point communication strategy between the master and each slave processing unit in an asynchronous implementation of PPSO for heterogeneous computing conditions. This condition happens, for example, when the number of parallel sub-swarms can not be equally distributed among the available processors. In this type of condition, a load balance technique is essential for the robustness of the algorithm.

The results obtained by Koh et al. [75] were compared with the algorithm presented by Schutte et al. [73], and showed that the asynchronous implementation performs better, in terms of parallel efficiency, when a large number of processors are used.

In 2007, McNabb et al. [76] introduced the MapReduce function for the PPSO. This function has two sub-functions: map and reduce.

On the one hand, the map function finds a new position, computes the velocity of the particle, evaluates the objective function on its position, updates the information of the personal best position and shares this information among all dependent particles. On the other hand, the reduce function receives the information and updates the global best position information.

This type of formulation allows the algorithm to be split into small procedures and easily balanced and scaled across multiple processing units, following the divide-and-conquer parallel approach.

Aljarah and Ludwig [77] proposed a PPSO optimisation clustering algorithm (MR-CPSO) based on the MapReduce approach. This parallel PSO-based algorithm showed efficient processing when large data sets were used.

Han et al. [78], in turn, included constraint handling in PPSO, whereas Gülcü and Kodaz [79] proposed a synchronous parallel multi-swarm strategy for PPSO.

In this multi-swarm approach, a population is divided into subpopulations: one master-swarm and several slave-swarms which independently run a PSO variant. However, the slave-swarms cannot communicate with each other, since communication is made through the master-swarm by migrating particles. The parallel multi-swarm algorithm also uses a new cooperation strategy, called Greed Information Swap [79]. This work was extended by Cao et al. [80] to include multi-objective optimisation.

Lorion et al. [81], in turn, proposed an agent-based PPSO that splits PPSO into sub-problems. There are two types of agents: one coordination agent and several swarm agents, which, similarly to the multi-swarm strategy, do not communicate with each other.

Then, a strategical niching technique is used to increase the quality gain. A fault tolerance (e.g., when a processing unit stops responding to requests) was also implemented, by either saving agent’s state in other swarm agents or by using the coordination agent’s information available at the moment about the failed agent.

Along with all these developments, some researchers suggested approaches that used a GPU instead of using a CPU, especially when the CUDA development kit of NVIDIA was released. GPUs are designed for image processing and graphics applications, although they have more processing capacity (since they have more processing elements) than CPUs.

Developing parallel algorithms on a GPU is far more complicated than the corresponding implementations on a CPU [82]. However, several studies have reported significant improvements in terms of execution time when a GPU implementation of the PPSO is compared to its corresponding implementation on a CPU (see, e.g., [83,84,85,86]).

A GPU-based fine-grained PPSO was proposed by Li et al. [59]. In turn, the performance of the Euclidean PSO, proposed by Zhu et al. [87], was improved by Dali and Bouamama [88], where a GPU-based parallel implementation of the original algorithm was presented.

Finally, it is also worth mentioning the distributed and load balancing versions of the PSO algorithm on GPU developed by using a grid of multiple threads [89] or distributed memory clusters [90], along with the OpenMP API.

## 5. Connections to Other Artificial Intelligence Tools

### 5.1. Hybrid Variants of Particle Swarm Optimisation

A PSO variant is called hybrid when the PSO algorithm is combined with other optimisation techniques, such as the operators used in GA (e.g., selection, crossover/recombination and mutation) and other population-based algorithms.

The objective of hybridization is to increase the quality of particles in a swarm and improve the effectiveness and efficiency of the algorithm. The PSO algorithm is known by its tendency to become trapped in local optima, which prevents it from exploring other regions of the search space. Combining PSO with other EA can overcome this difficulty in escaping from local optimal solutions and suppress the inherent deficiencies of other algorithms with which it is hybridised.

#### 5.1.1. Evolutionary Computation Operators

In 1998, Angeline [91] incorporated a selection mechanism into PSO similar to those used in more traditional evolutionary algorithms, thereby producing what is considered the first hybrid PSO algorithm.

That mechanism compares the current fitness of each particle with the fitnesses of other particles, and the least fit score a point. Then, the population is sorted using this score.

Current positions and velocities of the worst half of the population are then replaced with the positions and velocities of the best half, leaving the personal best position unchanged. Thus, the selection process resets the low-scored particles to locations within the search space that have yielded better results.

It was shown that this truncation selection mechanism incorporated into PSO improves the performance of the algorithm significantly on most of the tested functions. The roulette wheel selection operator was also used by Yang et al. [92], wherein the best particles in the swarm are the more likely to be selected.

On the other hand, many researchers then suggested and reported good performance by combining PSO with crossover operators (see, e.g., [4,34]) and different mutation strategies, such as Gaussian and Cauchy mutations [32,93,94,95]. These researches were essentially motivated by the fact that PSO presents difficulty in finding optimal or near-optimal solutions for many complex optimisation problems, including multimodel function optimisation and multi-objective optimisation.

Mutation is a genetic operator, analogous to the biological mutation, which, with a certain probability, changes the value of ${\vec{g}}_{\;t}$ or the next particle’s position from its current state, hoping to find a better solution, while maintaining the population diversity. This operation provides strong exploration and exploitation capabilities to the swarm and also prevents premature convergence to a local optimum.

For example, the Cauchy mutation operator can be implemented as follows [93]:

(31) $${\left({\vec{g}}_{\;t_{1}}\right)}_{d}={\left({\vec{g}}_{\;t}\right)}_{d}+\left({\left({\vec{x}}_{max}\right)}_{d}-{\left({\vec{x}}_{min}\right)}_{d}\right)\times \mathrm{Cauchy}\left(0,\sigma \right),$$

where ${\left({\vec{g}}_{\;t}\right)}_{d}$ and ${\left({\vec{g}}_{\;t_{1}}\right)}_{d}$ are, respectively, the current and the new values of the global best position for dimension *d*, and ${\left({\vec{x}}_{max}\right)}_{d}$ and ${\left({\vec{x}}_{min}\right)}_{d}$ are the upper and lower limits of the dimension *d*. Finally, σ is the scale parameter of Cauchy mutation, which is updated as follows:

(32) $$\sigma_{t+1}=\sigma_{t}-\frac{1}{t_{\max}}.$$

As can be seen, σ linearly decreases at each iteration, so that, in the first iterations, the exploration capability is stronger, while in the last ones the exploitation ability is privileged. Naturally, this mutation operator can be applied to both gbest and lbest models, and often $\sigma_{0}=1$.

On the other hand, reproduction or breeding is the process of combining any two particles (chosen among the particles selected for breeding at a given breeding probability) and performing a crossover operation that generates two new particles based on the characteristics of their parents (which are replaced by those new particles). In their hybrid algorithm, Løvbjerg et al. [4] used an arithmetic crossover operator, so the position of each new child particle is computed as follows:

(33) $$\begin{array}{cc} {{\vec{x}}_{c_{1}}^{\;\;\;i}}_{\;\;t} & =r\times {{\vec{x}}_{p_{1}}^{\;\;\;i}}_{\;\;t}+\left(1-r\right)\times {{\vec{x}}_{p_{2}}^{\;\;\;i}}_{\;\;t}\;,\\ {{\vec{x}}_{c_{2}}^{\;\;\;i}}_{\;\;t} & =r\times {{\vec{x}}_{p_{2}}^{\;\;\;i}}_{\;\;t}+\left(1-r\right)\times {{\vec{x}}_{p_{1}}^{\;\;\;i}}_{\;\;t}\;, \end{array}$$

where *r* is a uniformly distributed random value between 0 and 1, and the velocities are given by [4]:

(34) $$\begin{array}{cc} {{{\vec{V}}_{\;\;c_{1}}}^{\;\;i}}_{\;\;t} & =\frac{{{\vec{V}}_{\;\;p_{1}}^{\;\;i}}_{\;\;t}+{{\vec{V}}_{\;\;p_{2}}^{\;\;i}}_{\;\;t}}{\left|{{\vec{V}}_{\;\;p_{1}}^{\;\;i}}_{\;\;t}+{{\vec{V}}_{\;\;p_{2}}^{\;\;i}}_{\;\;t}\right|}\left|{{\vec{V}}_{\;\;p_{1}}^{\;\;i}}_{\;\;t}\right|,\\ {{\vec{V}}_{\;\;c_{2}}^{\;\;i}}_{\;\;t} & =\frac{{{\vec{V}}_{\;\;p_{1}}^{\;\;i}}_{\;\;t}+{{\vec{V}}_{\;\;p_{2}}^{\;\;i}}_{\;\;t}}{\left|{{\vec{V}}_{\;\;p_{1}}^{\;\;i}}_{\;\;t}+{{\vec{V}}_{\;\;p_{2}}^{\;\;i}}_{\;\;t}\right|}\left|{{\vec{V}}_{\;\;p_{2}}^{\;\;i}}_{\;\;t}\right|. \end{array}$$

In the last two equations, the subscript *c* indicates the position or velocity of a child particle, while the subscript *p* identifies a parent particle.

These evolutionary computation operators aim to reduce the diversity loss in the swarm and can be combined with others. Despite usually slowing down the efficiency of the algorithm, they can produce better results, especially when faced with multimodal functions.

In 2002, Miranda and Fonseca [96] proposed an approach, denoted Evolutionary PSO (EPSO), which merged the concepts of evolutionary computation with PSO. In their algorithm, the operations of replication (where each particle is replaced *r* times; usually r=1), mutation (on the cognitive, social, and inertia weights), crossover and selection (before evaluation) were used to generate diversity and to enable the fittest particle to survive and propagate. This is analogous to the mechanism of survival of the fittest of natural selection, from the Darwinian theory of evolution [97].

Wang et al., in 2013, proposed the Diversity Enhanced PSO with Neighborhood Search (DNSPSO) [98], a PSO variant that includes a new diversity enhanced mechanism using a crossover operation, and a new neighbourhood search strategy.

The crossover operation is applied to each dimension of the current particle’s position, by replacing it with the correspondent previous dimension where the particle was in the search space. This operation is, however, applied according to a uniform random number within the range [0,1] generated for each dimension. If the generated random number is lower than a predefined probability, the particle’s position is recombined with the previous dimension. Otherwise, it remains unchanged.

This operation creates what those authors called a trial particle that replaces the current particle only if its fitness value is lower than the current fitness (for minimisation problems).

In turn, the neighbourhood search strategy interestingly combines the gbest and lbest models, creating two more trial particles, based on the gbest and the lbest information. This search strategy is applied according to a predefined probability, and it was developed to improve the exploration of the search space by the particles in the swarm.

Then, the current particle is replaced by the most fitted particle among the current particle, the trial particle derived from the gbest information and the one from the lbest information.

The results presented by those authors showed that the DNSPSO algorithm achieved better results when compared to other PSO variants, both in terms of the quality of the solutions found and performance.

#### 5.1.2. PSO with Genetic Algorithms

On the other hand, PSO was also combined with GA. In GA, similarly to PSO, there is a population of potential candidate solutions. Each element of the population has chromosomes that are mutated, based on a certain probability, to maintain a certain level of population diversity and improve the solution.

Each iteration is called a generation, and the algorithm reflects the process of natural selection, wherein the best fit individuals are chosen for reproduction in order to produce the next generation (which is expected to be better than the previous one).

PSO is known for not being able to effectively avoid being trapped in local optima during the search process. However, the GA algorithm can be used, along with its operators, to reduce this weakness.

On the other hand, GA has a slower convergence speed when compared with PSO [92,99]. These advantages and disadvantages motivated the researchers to develop optimisation algorithms that combine PSO with GA.

Robinson et al. [99] introduced the first hybrid approach using PSO and GA for optimisation of a profiled corrugated horn antenna.

In their approach, they used the result of the execution of one of the algorithms as a starting point to the other. They either first use PSO and then GA (PSO-GA), or vice-versa (GA-PSO).

When the solutions found by one of the algorithms show no improvement, the algorithm is changed to either PSO or GA.

Some other applications using PSO combined with GA were suggested to, e.g., recurrent network design [100], wherein individuals in a new generation are created by crossover and mutation operations as in GA, but also by running an instance of PSO.

However, unlike the previous approach, GA and PSO both work with the same population. In each generation, after the fitness values are computed, the top 50% of elements are marked for maturing (and the other half is discarded).

The maturing technique, handled by the PSO algorithm, is used to enhance the best-performing elements, instead of using them directly to reproduce and generate the next generation.

Parents are then chosen based on a tournament selection, and then crossover and mutation are applied to produce the next offspring.

Yang et al. [92] suggested a PSO-based hybrid combining PSO with the genetic operations of selection, reproduction, crossover and mutation.

Like the previous approach, the same population is used as input for the GA and PSO algorithm, but the enhancement of the population is done by applying the motional behaviour of the PSO algorithm, while the population diversity is maintained by the genetic mechanisms (selection, reproduction, crossover and mutation). Additionally, they showed the application of the algorithm to solve three unconstrained optimisation problems and three COP.

Valdez et al. [101] tried to integrate the results given by the PSO algorithm and GA by using fuzzy logic. In their approach, a fuzzy system is responsible for choosing, according to the last results of the execution of either the GA or the PSO algorithm, which one should be executed next. Besides that, other two fuzzy systems are also used, one to change the crossover probability and the mutation rate of the GA, and the other to adjust the cognitive and social acceleration factors of PSO.

They compared the hybrid variant with the individual GA and PSO approaches, and the hybrid algorithm was shown to be superior to the individual evolutionary methods.

Some hybrid variants of the PSO algorithm with GA were used, e.g., for cancer classification [102], route planning [103], task allocation and scheduling [104,105] and image classification [106].

#### 5.1.3. PSO With Differential Evolution

Differential Evolution (DE) also belongs to the class of evolutionary computation methods. Like PSO, DE tries to optimise a problem by iteratively improving a candidate solution (called agent, that belongs to a population of candidates) using meta-heuristics.

In addition, this method does not require that the functions involved are differentiable, and it was designed to solve optimisation problems with real-valued parameters.

Although it is not guaranteed that an optimal solution is ever found, it has a great ability to maintain an adequate level of diversity within the population, and to perform a local search in specific areas of the search space. However, it has no mechanism to memorise the previous process, so the combination of DE and PSO is promising.

Each agent is represented by a set of real numbers (the parameters of the objective function) and moves around in the hyperplane until a stopping criterion (e.g., accuracy or number of iterations) is satisfied.

DE uses mutation and crossover (using three different agents) for generating a new trial parameter vector. If the new parameter vector is better than the previous one when evaluated in the objective function, the newly generated vector replaces the current vector [107,108], in accordance with the principle of the survival of the fittest [97].

Hendtlass [109] proposed the first hybrid approach using PSO and DE. In his simple approach, the PSO algorithm runs conventionally, and from time to time the DE algorithm takes place to move the particles to better positions.

Two years later, Zang and Xie proposed the DEPSO algorithm [110]. In this case, PSO and DE run alternately according to the number of the current iteration. If the current iteration number is odd, then PSO runs; if is even, then DE is executed (or the other way around).

Additionally, the algorithm uses a bell-shaped mutation and crossover to increase the population diversity, but instead of applying both changes at the same time (as DE originally does), different operations are applied at a random probability.

Several applications of this hybrid algorithm based on PSO and DE have emerged, including digital filter design [111], multimodal image registration [112] and data clustering [113].

In 2003, inspired by EPSO, Miranda and Alves [114] proposed the Differential Evolutionary PSO (DEEPSO), an algorithm that is similar to the EPSO sequence, but in which the velocity of each particle is calculated as:

(35) $${\vec{V}}_{\;t+1}^{\;i}=\omega {\vec{V}}_{\;t}^{\;i}+\varphi_{1}{R_{1}}_{\;t}^{\;i}\left({\vec{x}}_{\;t}^{\;r}-{\vec{x}}_{\;t}^{\;i}\right)+\rho \left(\varphi_{2}{R_{2}}_{\;t}^{\;i}\left({\vec{g}}_{\;t}^{\;*}-{\vec{x}}_{\;t}^{\;i}\right)\right),$$

where ρ is a diagonal matrix with 0 s and 1 s that controls the flow of information within the swarm (and can be seen as defining the communication topology among particles). ${\vec{x}}_{\;t}^{\;r}$ is a distinct particle from ${\vec{x}}_{\;t}^{\;i}$ that belongs to the set of particles currently in the search space or from the previous best particles, and can be chosen at random in the current iteration and be the same for all particles or different for each one.

Finally, ${\vec{g}}_{\;t}^{\;*}$ is given by:

(36) $${\vec{g}}_{\;t}^{\;*}={\vec{g}}_{\;t}\left(1+w_{g}N\left(0,1\right)\right),$$

where $w_{g}$ is a parameter or weight in the form of a diagonal matrix to add noise to the best position in the swarm, and N(0,1) is the standard normal distribution.

Those authors suggest that ${\vec{g}}_{\;t}^{\;*}$ can be chosen from the past bests and sampled once from ρ or can be sampled from ρ to each particle, although, according to the results presented, sampling ${\vec{g}}_{\;t}^{\;*}$ from past bests to each particle leads to the best results.

Other hybrid approaches using PSO and DE have been proposed. These include, for example, the LEPSO algorithm, developed by Abdullah et al. [115] with the objective of improving local best particle searching; and the enhanced DEPSO with adaptive parameters for the position update equation presented by Omran et al. [116]. On the other hand, Pant et al. [117] incorporated the PSO algorithm in DE to create a perturbation in the population that helps maintain diversity within the population and produce a good optimal solution; meanwhile, Epitropakis et al. [118], in addition to the social and cognitive experience of the swarm, included the personal experience of each particle in their hybrid approach.

Zhang et al. [119] used PSO and DE alternately, including the lbest and gbest models of the PSO algorithm. Xiao and Zuo [120] used a multi-population strategy in the DEPSO algorithm to improve diversity and keep each subpopulation on a different peak. In turn, Omran [121] presented a DE-PSO algorithm with a constriction factor, whereas Das et al. [122] used an hybrid approach of these algorithms combined with some concepts of SA, such as the probability of accepting poor solutions.

Either way, these authors did not explore the possibility of executing the PSO in the initial iterations and a change coming to the DE algorithm at the final stages of the algorithm, wherein local search around a potential solution to a problem is more advantageous.

#### 5.1.4. PSO with Simulated Annealing

SA is also a meta-heuristic optimisation algorithm which is based on the thermodynamic process of annealing; that consists of the slow and controlled cooling of a metallic material, in order to alter its microstructure, and with this, change and improve its main properties, such as strength, hardness and ductility. This process ends when the material reaches a state of minimum energy.

As other meta-heuristics, SA does not make any assumption on the continuity, differentiability or convexity of the cost and constraint functions of the problem. However, unlike PSO, SA accepts poor solutions by a given probability to maintain the diversity and improve the search process [123].

SA incorporates an important mechanism called cooling schedule, which controls the decreasing of temperature during the optimisation process and the deteriorations in the objective function.

At the very beginning, the annealing process (and SA) requires higher temperatures. Then, the temperature is decreased, and some candidates are generated at that temperature level.

A candidate solution is accepted when its fitness value is lower than the current configuration (for minimisation problems). Otherwise, it may still be accepted with a certain probability, but as temperature decreases only small deteriorations are accepted.

This strategy allows uphill moves that help SA to escape from optimal local solutions towards the end of the algorithm, when no deteriorations of the objective function are accepted.

Hybrid variants of PSO and SA were proposed because of the well-known inability of the PSO algorithm to jump out of local optima, and because the SA algorithm is known for making uphill movements and escaping from those solutions, avoiding premature convergence. Unfortunately, this does not ensure that the algorithm can always converge to the global minimum. Besides that, the computational effectiveness and efficiency of these hybrid algorithms can also be compromised.

The first studies on a hybrid algorithm based on PSO and SA were made by Wang and Li [124], Zhao et al. [125] and Yang et al. [123]. Wang and Li showed that, after evaluating each particle’s fitness, running SA independently on each of them and changing the movement according to the PSO algorithm can speed up the rate of convergence and enable the algorithm to escape from local optimal solutions. The algorithm was named SAPSO [124].

Zhao et al. [125] proposed the HPSO algorithm, in which the PSO runs first, providing an initial solution for SA during the hybrid search process. On the other hand, the PSOSA algorithm, proposed by Yang et al. [123], runs the PSO and the SA algorithm simultaneously; that is, after computing the velocity and position of each particle in the swarm using Equations (2) and (6), a Gaussian mutation operation is applied on each particle’s personal best position. If the new value found is lower than the previous one (in the case of a minimisation problem), then it is replaced by this new value; otherwise, the solution can still be accepted according to a certain probability. A similar algorithm was proposed by Sadati et al. [126].

Both hybrid algorithms showed to be successful when compared to the PSO algorithm and the SA algorithm separately, in terms of search behaviour (and thus the quality of the solutions found), performance and computation speed.

Xia and Wu [127] proposed another hybrid approach combining PSO and SA, in this case for the job-shop scheduling problem. Like in HPSO, in this hybrid algorithm PSO provides an initial solution for SA. Chu et al. [128], in turn, proposed a parallel PSO algorithm with adaptive SA (ASA-PPSO).

PSO algorithms with SA were also used by Shieh et al. [129] and Deng et al. [130], in which the Metropolis criterion was used to determine the acceptance of a new-found solution that is worse than the previous one. A hybrid discrete PSO-SA algorithm was proposed by Dong et al. [131] for the optimal elimination ordering problem in Bayesian networks. In turn, He and Wang [132] suggested a hybrid approach involving PSO and SA for constrained optimisation problems, which applies SA to the best solution of the swarm to help the algorithm in escaping from local minima.

#### 5.1.5. PSO With Other Evolutionary Algorithms

GA, DE and SA were not the only meta-heuristics that were combined with PSO.

In the literature (see, e.g., [17]), it is possible to find PSO-based hybrid algorithms that use, for example, Ant Colony Optimisation (ACO) (e.g., [133]), a population based meta-heuristic algorithm inspired by the social behaviour of real-life ants searching for food; Cuckoo Search [134], a meta-heuristic approach idealised to reproduce the breeding behaviour of cuckoo birds, who leave their eggs in the nests of other host birds of different species; and Artificial Bee Colony (ABC) optimisation [135], a swarm-based meta-heuristic algorithm based on the behaviour of real honey bee colonies, which are organised in groups of bees to maximise the nectar amount found in a food source.

It is important to note here that, considering the large number of new developments in this field, especially in the last decade, only the hybrid PSO-based algorithms that are most relevant in practice or future research have been addressed and emphasised in this section.

### 5.2. Artificial Neural Networks with Particle Swarm Optimisation

The first experiment on using PSO to train Artificial Neural Network (ANN) weights was made by Eberhart and Kennedy in the two papers that introduced PSO [1,2].

They claimed to have successfully trained a feedforward multilayer perceptron ANN using PSO to solve the exclusive OR (XOR) problem and to classify the Fisher’s Iris data set, which lead to the same, and sometimes better, results as the backpropagation algorithm.

It should be noted that the inertia weight is similar to the momentum term in a gradient descent ANN training algorithm [37].

Eberhart and Hu [136] showed the use of sigmoid activation functions in training a feedforward multilayer perceptron ANN using PSO to classify tremor types in Parkinson’s disease.

They used an ANN with 60 inputs, 12 hidden nodes and two outputs nodes. Despite the small size of the data set, PSO has been successfully applied to train the ANN with low error and high performance.

In turn, Engelbrecht and Ismail [137] showed that the PSO could also be used to train product unit ANNs (in which the output of each node is computed as a weighted product), and when compared to other training algorithms, such as GA, the PSO showed the lowest errors.

Kennedy [55] used the social-only and the cognition-only models to train an ANN for solving the XOR problem, and showed that the social-only version outperformed the cognition-only model.

The cooperative learning approach presented in Section 4.1 was used by van den Bergh and Engelbrecht [36], and different two-layered network architectures were considered for testing; namely, plain (where a single swarm was used to train all the weights and bias), Lsplit (in which two swarms were used to train each layer), Esplit (where one swarm optimised 90% of the weights and the other swarm optimised the remaining) and Nsplit (similar to Esplit, but in which weights were split according to a function).

These authors performed some tests on various databases, and split architectures (especially the Esplit architecture) outperformed the plain architecture in terms of performance, although correlated variables should be removed of the data set beforehand to improve the effectiveness of these type of architectures.

Zhang and Shao [138] split the data set into three sets, a training set, a validation set and a testing set, and used the PSO to train the architecture of ANN, including the number of nodes, generated at algorithm initialisation.

Chatterjee and his collaborators [139] showed that the PSO algorithm can be used to train the weights of a Takagi–Sugeno neuro–fuzzy network for voice-controlled robot systems.

A detailed comparison of PSO and backpropagation as training algorithms for ANN was made by Gudise and Venayagamoorthy [140]. Results showed that the ANN’s weights converge faster with the PSO than with the backpropagation algorithm to achieve the same error goal.

On the other hand, Mendes et al. [141] showed that, for the problems they considered, PSO is not the best algorithm for ANN training, but it is the best one when a high number of local minima is known to exist.

Juang [100] applied PSO to recurrent neural/fuzzy network training, by combining GA, PSO and the concept of elite strategy to produce the best network design.

Ince et al. [142] used a modified version of the PSO algorithm, called MD PSO, to find the optimal architecture and weights of a feedforward multilayer perceptron ANN for the classification of electrocardiogram signals.

The MD PSO algorithm was proposed with the aim of finding the optimal solution in the search space, but also the best number of dimensions for that search space; that is, the particles explore the search space with different dimensions, and at the end of the algorithm, the global optimal solution is chosen according to the lowest optimal solution found from each dimension.

Interestingly, a hash function was used to set higher hash indexes to ANNs with higher complexity, i.e., with higher numbers of hidden layers and neural units per hidden layer, and thus the MD PSO can be used to optimise this unique dimension and find the simplest ANN that is able to correctly classify electrocardiogram signals.

It was also shown by Ince et al. [142] that the proposed algorithm strategy performs better than most of the existing algorithms for classification of electrocardiogram patterns.

Pehlivanoglu [143], in turn, used a periodic mutation strategy to determine which particles should be mutated, when the operation should happen, and which ones should be added to the swarm.

Quan et al. [144] also integrated mutation in the PSO algorithm to train a feedforward ANN to short-term load and wind power forecast.

Besides optimising the network architecture and the weights of each connection, Garro et al. [145] also computed the best transfer (or activation) function for the problems at hand.

Al-Kazemi and Mohan [146] used the Multi-Phase PSO (MPPSO) algorithm [147] with ANN. This variant of the PSO algorithm uses niching techniques to increase the diversity and the exploration of the search space. Besides that, according to the phase of PSO execution, the direction of each particle changes, and the particles only move to positions that will increase their fitness [148].

When compared with the backpropagation algorithm, MPPSO showed to be the more stable algorithm for optimising the ANN weights for the problems considered.

Conforth et al. [149], on the other hand, used a hybrid PSO approach, combining PSO and ACO, to adjust the ANN connection weights for the XOR problem.

In the aforementioned approaches, the PSO algorithm and its variants are used for ANN training. The use of the backpropagation algorithm for network training is neglected, since, in addition to requiring gradient and differentiable information, it also suffers from slow convergence and has a high probability of getting trapped in local minima when compared with PSO [150,151].

Furthermore, in most of these approaches, PSO seems to need fewer epochs to get good results when compared to the backpropagation algorithm.

**Figure 3:**
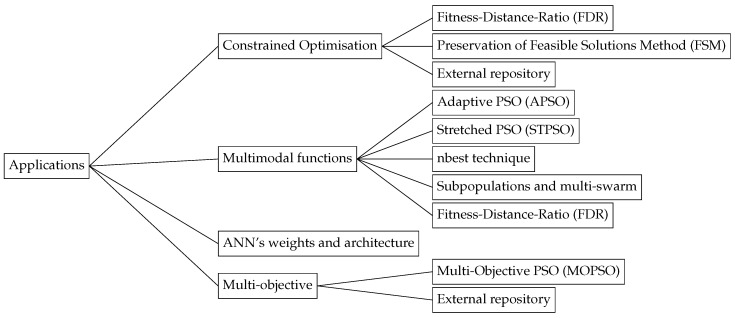
Summary of the most important applications of pso.

As can be seen in the previous sections, PSO is one of the most used metaheuristic optimisation algorithms, and is currently being applied for different purposes, as can be seen in Figure 3.

## 6. Conclusions

In the previous sections, a literature review focusing on the PSO algorithm and its variants was presented, describing the most important developments in this field since the introduction of the algorithm in mid-1990s.

The PSO algorithm was inspired by some characteristics of the collective behaviour observed in the natural world, in which elements of a population cooperate with each other seeking to obtain the greatest mutual benefit.

Over the years, the PSO algorithm has gained attention from many researchers due to its simplicity and because it does not make assumptions on the characteristics and properties (such as continuity or differentiability) of the objective function to be optimised.

Inevitably, the algorithm has suffered changes to, for example, improve its effectiveness and efficiency.

The use of different topologies was one of the first suggestions to improve the algorithm. However, a conclusion was reached: the topologies of communication are problem-dependent.

PSO was widely used for different applications, which led to some researchers to report convergence problems with the algorithm. To lessen this problem, changes were made, mostly by the introduction of new parameters, or by combining PSO with other operators or algorithms.

The algorithm has been also extended to solve a panoply of different problems and applications since its original formulation in 1995. Constrained, multi-objective and multimodal optimisation problems were some of the most relevant applications and problems solved with the PSO approach.

To conclude, PSO is one of the leading swarm intelligence algorithms and is superior when compared to other optimisation algorithms in some fields of application. Although it has some drawbacks, those were lessened by using different types of strategies and modifications to the original version of the algorithm. PSO is also a problem-independent algorithm; i.e., it can be used in a wide range of applications due to its great capacity for abstraction, which further highlights its importance.

## Abbreviations

The following abbreviations are used in this manuscript:

PSO	Particle Swarm Optimisation
GA	Genetic Algorithms
ANN	Artificial Neural Network
FSM	Preservation of Feasible Solutions Method
GCPSO	Guaranteed Convergence PSO
FIPS	Fully Informed Particle Swarm
STPSO	Stretched PSO
APSO	Adaptive PSO
DE	Differential Evolution
EA	Evolutionary Algorithms
SA	Simulated Annealing
PPSO	Parallelized PSO
CPU	Central Processing Unit
GPU	Graphics Processing Unit
COP	Constrained Optimisation Problem
FDR	Fitness-Distance-Ratio
MPPSO	Multi-Phase PSO
SPSO	Standard PSO
CPSO	Cooperative PSO
MOPSO	Multi-Objective PSO
SCD	Special Crowding Distance
EPSO	Evolutionary PSO
DEEPSO	Differential Evolutionary PSO
ACO	Ant Colony Optimisation
DNSPSO	Diversity Enhanced PSO with Neighborhood Search
ABC	Artificial Bee Colony

## Mathematical notation

*D*	Mean distance of each particle to other particles
F(·)	Penalty function to be minimised or maximised
H(·)	Function stretching for multimodal function optimisation
$H_{p}\left(\cdot \right)$	Penalty factor in a penalty function
K(·)	Constriction factor
$N_{i}$	Neighbourhood of the particle *i*
$R_{1}$	Cognitive uniformly distributed random vector used to compute the particle’s velocity
$R_{2}$	Social uniformly distributed random vector used to compute the particle’s velocity
*S*	Search space, defined by the domain of the function to be optimised,
	that contains all the feasible solutions for the problem
α	Diagonal matrix whose diagonal values are within the range of [0,1]
ϵ	Absolute different between the last and the current best fitness value, or the algorithm accuracy
γ(·)	Power of a penalty function
$\hat{y}$	Position of the best particle in the swarm or in the neighbourhood (target particle)
ω(·)	Inertia weight parameter used to compute the velocity of each particle
ρ	Diagonal matrix that represents the architecture of the swarm
σ	Scale parameter of Cauchy mutation
τ	Index of the global best particle in the swarm
θ(·)	Multi-stage assignment function in a penalty function
$\varphi_{1}$	Cognitive real acceleration coefficient used to compute the particle’s velocity
$\varphi_{2}$	Social real acceleration coefficient used to compute the particle’s velocity
$\varphi_{3}$	Deviation real acceleration coefficient used to compute the particle’s velocity
${\vec{P}}_{\;t}^{\;j}$	Prior best position that maximises the FDR measure
$\vec{V}$	Particle’s velocity
${\vec{c}}^{\;j}$	Position of the centroid of the group *j*
$\vec{g}$	Global best position of a particle in the swarm
${\vec{p}}_{\;t}^{\;i}$	Personal best position of particle *i*
$\vec{x}$	Position vector of a solution found in the search space
${\vec{x}}_{max}$	Upper limit of the dimension *d* in the search space
${\vec{x}}_{min}$	Lower limit of the dimension *d* in the search space
$\vec{y^{*}}$	Set of feasible solutions that forms the Pareto front
*d*	Number of dimensions of the search space
$e_{f}$	Evolutionary factor used in the APSO
f(·)	Objective function to be minimised or maximised
*g*	Set of inequality function constraints
*h*	Set of equality function constraints
$h_{p}\left(\cdot \right)$	Dynamic modified penalty value in a penalty function
*l*	Number of particles in the swarm or in the neighbourhood
*m*	Number of inequality constraints
*n*	Non-linear modulation index
*p*	Number of equality constraints
$q_{i}\left(\cdot \right)$	Relative violated function of the constraints in a penalty function
*s*	Number of particles in the swarm
*t*	The number of the current iteration
$w_{g}$	Parameter, in the form of a diagonal matrix, to add variability to the best position in the swarm
